# A Benchmark for Physics-informed Machine Learning of Chlorine Concentration States in Water Distribution Networks

**DOI:** 10.1007/s42979-025-04008-y

**Published:** 2025-06-04

**Authors:** Luca Hermes, André Artelt, Stelios G. Vrachimis, Marios M. Polycarpou, Barbara Hammer

**Affiliations:** 1 Bielefeld University, Inspiration 1, 33615 Bielefeld, NRW Germany; 2 KIOS Research and Innovation Center of Excellence, University of Cyprus, Panepistimiou 1, Aglantzia, 2109 Nicosia, Cyprus; 3 Electrical and Computer Engineering Department, University of Cyprus, Panepistimiou 1, Aglantzia, 2109 Nicosia, Cyprus

**Keywords:** Water distribution networks, Water quality, Chlorine state estimation, Benchmark, Surrogate models, Deep learning, Graph Neural Networks, Recurrent Neural Networks

## Abstract

Ensuring high-quality drinking water is a critical responsibility of water utilities, with chlorine being the main disinfectant typically used. Accurate estimation of chlorine concentrations in the dynamic environment of water distribution networks (WDNs) is essential to ensure safe water supply. This work introduces a comprehensive and carefully created benchmark for training and evaluation of chlorine concentration estimation methodologies in WDNs. The benchmark includes a diverse dataset of 18,000 scenarios of the widely studied ‘Hanoi’, ‘Net1’, and the more recent and complex ‘CY-DBP’ water networks, featuring various chlorine injection patterns to capture diverse physical dynamics. To provide baseline evaluations, we propose and evaluate two neural surrogate models for chlorine state estimation: a physics-informed Graph Neural Network (GNN) and a physics-guided Recurrent Neural Network (RNN).

## Introduction

Water distribution networks (WDNs) are critical infrastructures that deliver treated water to consumers across vast, interconnected networks. Ensuring water quality in WDNs is essential for public health and safety, as contaminants can arise from natural sources, accidental pollution, or malicious actions, posing significant risks to human health. Monitoring water quality across these networks is challenging due to their large size and the various factors that influence water quality as it travels through the system [1]. Traditional water quality monitoring methods depend on a few fixed sensors strategically located within the network to detect changes in parameters such as chlorine residuals, pH, and total organic carbon (TOC). These sensors serve as early warning indicators of contamination; however, their limited coverage often leaves parts of the network unmonitored, potentially allowing contamination events to go undetected [2].

In recent years, advances in machine learning (ML) have enabled the development of data-driven methods for water quality estimation in WDNs. These approaches leverage multivariate data from sensor networks and predictive modeling techniques to improve real-time monitoring, event detection, and anomaly identification, even in parts of the network lacking direct sensor coverage [3]. Early work by Perelman et al. [4] demonstrated how artificial neural networks (ANNs) combined with sequential probability updates can effectively detect contamination events by analyzing multivariate time-series data from sensors. Following this, models using hybrid methods, such as combining ANNs with support vector machines (SVMs) and Bayesian updating, have proven effective for real-time anomaly detection by processing high-dimensional data and dynamically updating the probability of an event [5].

Chlorine is a widely used disinfectant by water utilities worldwide to prevent the growth of harmful bacteria in treated water as it moves through distribution networks. When maintained at prescribed concentrations, it ensures public health safety while effectively inhibiting microbial growth. Accurately estimating chlorine concentrations across the entire network, including locations without direct sensor coverage, is challenging due to the limited number of sensors and significant modeling uncertainties [6]. This task, known as dense state estimation, aims to predict chlorine residuals throughout the WDN based on limited sensor data and has thus emerged as a key area of research. Li et al. [7] used a gated graph neural network (GGNN) to perform spatio-temporal dense state estimation, predicting chlorine concentrations across a real-world WDN in China. By incorporating historical data, sensor readings, and topological information, their model achieved high predictive accuracy for chlorine levels at unmonitored locations. Salem et al. [8] developed a Topology Adaptive Graph Neural Network (TAGNN) for purely spatial chlorine estimation, predicting chlorine concentrations across the network based on sparse sensor data at the current time step. Evaluated on the C-Town benchmark [9], the TAGNN model effectively used network structure, although it did not include temporal dynamics or flow directionality.

Other approaches have focused on specific node estimation, predicting chlorine residuals at critical nodes based on temporal data. Riyadh et al. [10] investigated convolutional neural networks (CNNs) and long short-term memory networks (LSTMs) to predict chlorine residuals in the main nodes of the network. Using a real-world dataset from a WDN in Canada, they demonstrated how temporal modeling can effectively capture the non-linear, dynamic relationships among operational parameters and chlorine levels, achieving high accuracy.

Moreover, due to the effect that certain contaminants have on chlorine concentration, several methods have indirectly used the estimation of chlorine concentration for contamination detection. Li et al. [11] explored contamination detection using generative adversarial networks (GANs), leveraging spatial-temporal correlations across multiple sensor locations to detect anomalies. Tested on real-world and synthetic data, this method improved detection capabilities for a variety of contamination events, highlighting the advantages of combining spatial and temporal modeling in WDNs. Similarly, Tinelli and Juran [2] applied SVMs and ANNs to monitor multiple water quality parameters for bio-contamination, achieving robust results for early contamination detection in real-time scenarios.

However, the aforementioned approaches are not compared to other methods from the literature which is likely due to the fact that they address slightly different tasks and work on proprietary datasets. Consequently, comparing existing research on chlorine state estimation poses significant challenges due to the diverse datasets employed, encompassing both simulated and real-world data. These datasets are frequently not made publicly accessible, which hinders the ability of researchers to validate and replicate findings or to build upon previous work. The lack of standardized datasets and open access further complicates efforts to establish consistent evaluation metrics or benchmarks, leading to difficulties in assessing the relative performance of different methods and models. As a result, the advancement of knowledge in this field may be slowed down, underscoring the need for more open data-sharing practices and standardized evaluation frameworks.

*Our contributions* In this work, we introduce the first publicly accessible and open benchmark, comprising both a dataset and evaluation metrics, for developing chlorine state estimation methodologies for water distribution networks (WDNs). Additionally, we propose and assess two different deep neural networks – physics-guided Recurrent Neural Networks (RNNs) and physics-informed Graph Neural Networks (GNNs) – to serve as baselines for machine learning-based chlorine state estimation in the form of neural surrogate models.

## Foundations

### Water Quality Modeling

Water quality defines the suitability of water for consumption by humans and animals. It is determined by the concentration of specific key variables in the water. The dynamics of these concentrations describe how the mass per unit volume (concentration) of physical, chemical, and biological constituents (referred to as *species*) within the WDN vary across space and time.

Let $w \in {\mathbb {R}}^{n_w}$ be a vector indicating the concentration of $n_w$ substances within a WDN at a certain location and time, with $w^{(i)}$ for $i \in \{1, \dots , n_w\}$ corresponding to the concentration of the $i^{th}$ species. We are typically concerned with a subset of species that must be regulated due to their health impact. For instance, $w^{(1)}$ may represent the concentration of a disinfectant (e.g., Chlorine), and $w^{(2)}$ may represent bacteria (e.g., *E. coli*).

In general, multiple species may coexist within a WDN. Some react to form new chemical compounds, while others are non-reactive and may remain constant. Both types are diluted and transferred, changing concentration at different network locations over time. This constitutes a dynamical system governed by advection and reaction dynamics.

In the WDN, when substances enter a pipe (edge $e_{(i,j)}$), they are transported with the water flow. This change in substance concentration over space and time is called *advection* and is typically described by a first-order hyperbolic partial differential equation:

1 $$\begin{aligned} \frac{\partial {w}_{(i,j)}(z,t)}{\partial t} + \frac{q_{(i,j)}(t)}{\alpha _{(i,j)}} \frac{\partial w_{(i,j)}(z,t)}{\partial z} = 0, \end{aligned}$$

where $w_{(i,j)}(z,t)$ is the vector of substance concentrations at time $t$ and distance $z$ along pipe $e_{(i,j)}$ with flow $q_{(i,j)}(t)$ and cross-sectional area $\alpha _{(i,j)}$. Note that $\frac{q_{(i,j)}(t)}{\alpha _{(i,j)}}$ yields the water velocity in the respective pipe.

The reaction dynamics characterize how the concentration of one or more substances changes due to reactions, decay, or growth within a finite volume of water. These are typically described by single-species reaction dynamics, which model the rate of decay or growth for a single substance, aggregating the interactions with others.

For modeling multiple species, reaction dynamics in a static environment (i.e., neglecting the *z* dimension) involve coupled differential and algebraic equations:

2 $$\begin{aligned} \frac{dw(t)}{dt}&= f_r(w(t), \Theta _r), \end{aligned}$$

where $w \in {\mathbb {R}}^{n_w}$ is a vector of concentrations of $n_w$ substances, $f_r(\cdot )$ describes the concentration change due to decay/growth reactions, and $\Theta _r$ represents reaction kinetics. In practice, $f_r$ is derived from the stoichiometry of the reactions and may include linear or bilinear terms. A special case of (2) is the first-order decay model:

3 $$\begin{aligned} \frac{dw(t)}{dt}&= \theta _r w(t), \end{aligned}$$

where $\theta _r \in \Theta _r$ is a parameter vector indicating decay/growth rates for the substances in $w$.

Combining the advection and the reaction dynamics yields a PDE for water quality modeling that can be applied to a multi-species case:

4 $$\begin{aligned} \frac{\partial {w}_{(i,j)}(z,t)}{\partial t}&+ \frac{q_{(i,j)}(t)}{\alpha _{(i,j)}} \frac{\partial w_{(i,j)}(z,t)}{\partial z} = f_r(w_{(i,j)}(z,t), \Theta _r ) \nonumber \\&\quad + u^q_{(i,j)}(z,t) \nonumber \\&\quad + \phi _{(i,j)}(z,t), \end{aligned}$$

were $f_r(\cdot )$ denotes changes in concentration due to reactions, $u^q_{(i,j)}$ represents controlled inputs, such as added disinfectants, and $\phi _{(i,j)}(z,t)$ represents uncontrolled contamination inputs, such as accidental injections.

Water quality in tanks is often modeled using a Continuous Stirred-Tank Reactor (CSTR) approach [12], which assumes uniform mixing within the tank. For the $i^{th}$ tank $v_i \in {\mathcal {V}}_T$, the average concentration $x_i^q(k) = w_i(k)$ can be computed based on inflows, outflows, and reactions occurring within the tank. However, in this work, we omit the modeling of water quality within tanks as the primary focus is on the distribution network itself, where the dynamics of water quality and contaminant spread are more complex and directly relevant to end-user consumption.

Equation (4) represents physical constraints that can guide model predictions by incorporating known physical principles-such as chlorine decay and transport dynamics-ensuring that the learned models not only fit the data but also adhere to real-world water quality behaviors. Beyond replicating the system’s dynamics, additional physical constraints on parameter values are incorporated into these models. These include enforcing a minimum chlorine concentration (which must be greater than or equal to zero) and ensuring that the maximum chlorine concentration does not exceed the injection point concentration. Such constraints enhance model robustness and prevent physically unrealistic predictions.

### Recurrent Neural Networks

Recurrent Neural Networks (RNNs) are a type of neural network architecture distinguished by their ability to incorporate memory components, enabling them to effectively process and analyze sequences of data points, such as time series. This memory capability allows RNNs to retain information from previous inputs, making them particularly well-suited for tasks where context or historical data is important. Unlike traditional neural networks, which treat each input independently, RNNs leverage their recurrent connections to maintain a dynamic internal state, which is updated as each new data point in the sequence is processed. This characteristic makes them highly valuable in applications like natural language processing, speech recognition, and any domain where understanding the temporal dependencies and patterns within sequential data is crucial. However, RNNs can face challenges such as vanishing or exploding gradients during training, which has led to the development of advanced variants like Long Short-Term Memory (LSTM) networks [13] and Gated Recurrent Units (GRUs) [14] to address these issues and enhance their performance.

The central concept of RNNs is their memory which is usually realized by introducing a state vector $\vec {h}_t\in {\mathbb {R}}^k$ that is updated over time (by a parametrized function) as the sequence of data points is processed:

$$\begin{aligned} (\vec {x}_t, \vec {h}_t) \mapsto (y_t, \vec {h}_{t+1}) \end{aligned}$$

The aforementioned popular instances of RNNs such as LSTMs [13] and GRUs [14] mainly differ in the way the hidden state $\vec {h}_t$ is updated and how the output $y_t$ is computed.

### Graph Neural Networks

Graph neural networks (GNNs) [15–17] are gaining increasing popularity for applications in WDNs. GNNs have been successfully applied to hydraulic state estimation [18], surrogate modeling [19], and water quality estimation [7, 8]. One main motivator for this development is that WDNs are graph-structured domains. A WDN can be modeled as a graph by formalizing nodes (e.g. junctions, tanks, and reservoirs) as a set $V = \{v_1, .., v_N\}$, and pipes as edges ${\mathcal {E}} = \{ e_{ij} : \forall i \in V ; j \in {\mathcal {N}}(i) \}$. Here ${\mathcal {N}}(i)$ denotes the set of neighbors of node *i*. The edge $e_{ij}$ is directed from the sender node *i* to the receiver node *j*. The nodes are further specified by feature vectors $X = [{\textbf{x}}_1, ..., {\textbf{x}}_N]$ to encode attributes like demand, node type, and other features. The edge feature vectors $Q = [{\textbf{q}}_1, ..., {\textbf{q}}_{|{\mathcal {E}}|}]$ typically contain information on pipe lengths, diameters, roughness coefficients, and flows.

A variety of different GNNs exist, and many of them apply *message-passing*, a principle that can be understood as three successive operations: Message generation, message aggregation, and node update. These operations are implemented by neural networks. The $k^{th}$ GNN layer computes the node embedding for node *i* via

$$\begin{aligned} {\textbf{x}}_i^{(k)} = \gamma ^{(k)} \left( {\textbf{x}}_i^{(k-1)}, \sum _{j \in {\mathcal {N}}(i)} \, \phi ^{(k)}\left( {\textbf{x}}_i^{(k-1)}, {\textbf{x}}_j^{(k-1)},{\textbf{q}}_{j,i}\right) \right) , \end{aligned}$$

where $\phi ^{(k)}$ generates messages that are aggregated over the neighborhood ${\mathcal {N}}(i)$ by a permutation invariant aggregation function $\sum$, and $\gamma ^{(k)}$ computes the output embedding of node *i* after the $k^{th}$ layer.

#### GNN for Physics-Informed Machine Learning

The quality of water in WDS follows complex spatio-temporal dynamics, and data is often limited, especially in real-world scenarios, where only a small number of sensors is available. This scarcity of data in such a complex system makes machine learning models prone to overfitting. To develop models that generalize effectively, incorporating constraints and regularization techniques is often beneficial. One promising approach to achieve this is through the use of physical and chemical prior knowledge, which is the focus of physics-informed machine learning.

The basic principle of physics-informed machine learning is to use prior knowledge like constraints, symmetries, and governing equations to regularize machine learning models [20, 21]. These governing equations typically exist in the form of ordinary differential equations (ODEs) and PDEs. Several works have discovered the close similarity between the operations in a GNN and PDEs and adapted the message-passing operations to resemble specific existing PDEs (i.e., heat diffusion, advection) [22–24]. These types of models have been called continuous GNNs [25]. It has also been highlighted that many numerical schemes to solve PDEs are special cases of message-passing [26] as well, which is the main operation of a GNN layer. Consequently, GNNs have already been applied to multiple physical problems where physical knowledge is used as inductive biases to the model [19, 21, 27, 28]. These findings motivate the use of a GNN for water quality estimation—a system governed by advection and reaction dynamics that arise from local interactions.

In this work, we introduce a simple baseline GNN that learns a PDE as described in Sect. 3.3.2. To estimate chlorine concentrations we apply Euler integration to generate time series of chlorine levels. This presents an alternative approach to other temporal graph neural networks like gated graph neural network (GGNN) that was used to estimate water quality in [7]. Primarily, while GGNN handles temporal dynamics explicitly via gating mechanisms, the PDE GNN handles them implicitly in the continuous evolution of the state. In future work, the PDE GNN can be extended with the existing research of continuous GNNs and physics-informed machine learning.

## A Benchmark for Chlorine State Estimation

Currently, there do not exist any substantial benchmarks on the increasingly important problem of chlorine state estimation in water distribution networks (WDNs). We therefore propose an innovative benchmark designed to consistently evaluate and compare algorithms/methods for chlorine state estimation in WDNs by offering evaluation functions and comparisons. Furthermore, it also incorporates a framework to generate reasonable benchmarks such that researchers can easily create their own, modified, version of this proposed benchmark.

This benchmark offers considerable flexibility and can be applied to different tasks and configurations, including:

- Predicting chlorine concentration at a single node or at multiple nodes.
- Assuming full knowledge of all flows/chlorine concentrations, or only partial noisy knowledge, with the latter mimicking real-world use cases.

The entire benchmark, including the Python code for running simulations and generating the data, is publicly available on GitHub^1^ and on the WaterBenchmarkHub platform [29]^2^. The benchmark includes a dataset, along with a set of evaluation metrics, which both can be easily utilized in Python through the provided Python module. Comprehensive documentation accompanies both the benchmark and the Python modules to facilitate their use.

### Data Set

This benchmark contains scenarios for three different widely recognized Water Distribution Networks (WDNs) from the literature used for benchmarking: Net1, Hanoi, and the more recent and complex CY-DBP.

**Fig. 1:**
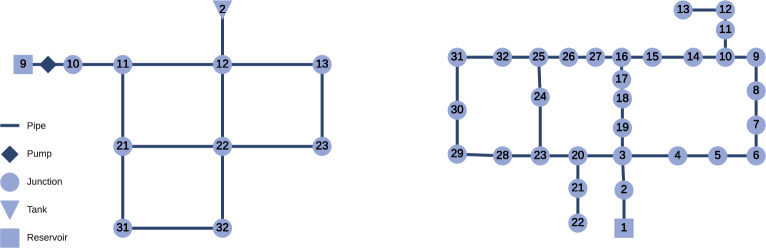
Two popular water distribution networks. Left: Net1; Right: Hanoi

For the Net1 and Hanoi network (refer to Fig. 1 for an illustration), we build on the LeakDB [30] benchmark – a benchmark dataset comprising numerous artificially generated yet realistic (leakage) scenarios, designed to facilitate the evaluation and comparison of leakage detection algorithms.

**Fig. 2:**
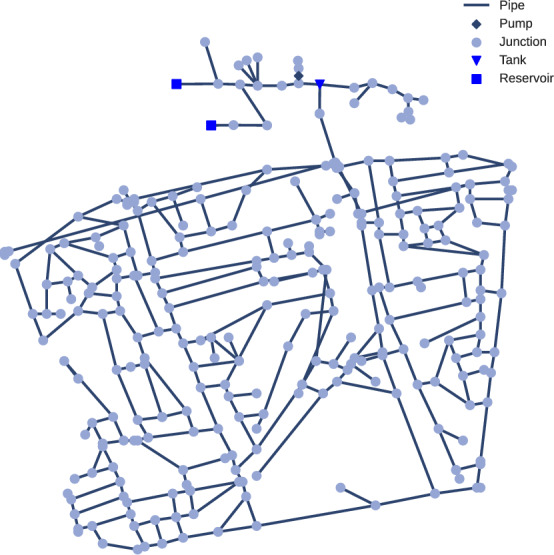
The CY-DBP water distribution network: 284 junctions, 357 pipes, 2 pumps, 4 valves, 2 reservoirs, and 1 tank. The two pumps are connected in parallel to the same nodes, the plot therefore shows only a single pump

For the CY-DBP network [31] (see Fig. 2 for an illustration), we mimic the uncertainties from LeakDB [30] to generate a large number of similar but slightly different scenarios.

The benchmark dataset in this work focuses on the transport, decay, and mixing of chlorine within water distribution networks, without explicitly modeling other substances. Its primary use case is chlorine state estimation, meaning it is intended to predict chlorine concentration at specific nodes or links in the network. The dataset is crafted to assess how effectively the physical processes—i.e. transport, decay, and mixing—are represented in a learned model. It includes scenarios designed for assessing adherence to underlying physical principles, such as those involving random demand patterns, which are artificially generated and hence easily scalable. The data set was created and simulated using the open-source Python package EPyT-Flow [32], which is based on EPyT [33] and EPANET [34]. As the quality dynamics depend on hydraulic dynamics [6], the hydraulics (e.g., flow rates, pressures, etc.) are calculated first, followed by the simulation of the quality dynamics.

For each of the three WDNs (i.e. Net1, Hanoi, and CY-DBP) the proposed benchmark includes 1000 scenarios, each lasting 30 days with 30-min time steps. These scenarios incorporate slightly varied demand patterns and network parameters, such as pipe lengths and diameters, to simulate uncertainties and evaluate the generalizability of the applied methods.

**Fig. 3:**
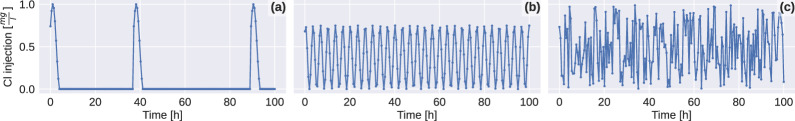
Different Chlorine injection patterns: **a** Spike pattern, **b** periodic Wave-like pattern, **c** completely random pattern

In all scenarios, a chlorine injection pump is placed at the network’s water inlet (reservoir), with each scenario featuring a separate instance for one of three distinct injection patterns illustrated in Fig. 3. Note that the CY-DBP network has two reservoirs, and therefore two chlorine injection pumps. Also, both the Net1 and the CY-DBP network contain tanks that introduce additional complexity in the dynamics. While the chlorine injection scenarios considered here are not representative of real-world practices, they enable empirical evaluation of adherence to physical principles. Successfully predicting chlorine states under these conditions demonstrates that the processes of transport, mixing, and decay have been effectively learned or modeled, as there are no extraneous correlations to rely upon. The injection patterns are designed as follows:

- **Spike**: Short bursts (i.e., spikes) of chlorine, as shown in Fig. 3a. The time intervals between impulses are randomized but always exceed the maximum transport delay (i.e., the time it takes chlorine to reach the most distant node). This pattern is particularly useful for understanding and learning substance transport times within the network, emphasizing advection dynamics.
- **Wave**: A periodic, wave-like pattern with a fixed but random frequency, illustrated in Fig. 3b. This pattern aids in learning chlorine decay rates, focusing on reaction dynamics.
- **Random**: A completely random pattern, depicted in Fig. 3c. This pattern is valuable for assessing both advection and reaction dynamics, offering a comprehensive test of the physical processes.

Furthermore, we also provide a version of each scenario where the demands have been randomized – i.e. breaking any potential correlations between demands and chlorine states.

The dataset comprises a total of 18,000 scenarios, providing information on the flow rate at each link, as well as the chlorine concentration at each node and link. Users have the flexibility to select only a subset of these quantities as input, simulating a specific sensor configuration. In this work however, we aim to replicate the simulator’s behavior by assuming full knowledge of the hydraulics, including flow rates, and the chlorine concentration only at the injection points (i.e., reservoirs). Our objective is to substitute the quality simulation with a neural surrogate model implemented by a deep neural network.

*Usage in Python* To enhance accessibility, a Python module for loading the dataset is also provided. This includes a general data loader and a specialized loader for use with PyTorch [35].
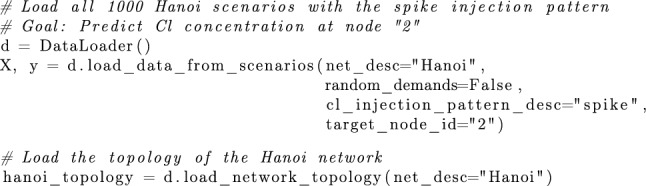


### Evaluation Metrics

We propose assessing performance using various, carefully chosen metrics. Here, we aim for easy-to-interpret metrics as well as metrics that are well-suited for the specific characteristics of the proposed benchmark. In particular, in the spike pattern (see Fig. 3a) where the ground truth chlorine concentration is zero most times, metrics such as the MAPE and $\hbox {R}^2$ would suffer from numerical issues (i.e. division by almost zero).

For a single node prediction, denoted as ${\hat{y}}_i$, this can be extended to multiple nodes by, for example, averaging over all nodes *i*:

- **Non-negativity of the predicted chlorine concentrations**—i.e. evaluating a trivial physical plausibility of the predicted concentrations:
  5 $$\begin{aligned} \sum _{t=1}^{T} \mathbbm {1}({\hat{y}}_i(t) \ge 0) \end{aligned}$$
- **Upper bound of a physically plausible chlorine concentration.** This depends on the maximum concentration at the injection points during past time steps, which may still influence the system. This concept is referred to as the memory of the system and relates to the maximum time required for water from the injection location to reach any node in the network. Given that flow rates are assumed to be known in this study, the maximum transport times for all nodes can be explicitly calculated. This also relates to the accuracy of predictions for each node, as a longer transport time implies a greater range of past inputs that can affect the output, introducing more uncertainty into the model.
  6 $$\begin{aligned} \frac{1}{T-K}\sum _{t=K}^T \mathbbm {1}\left( {\hat{y}}_i(t) \le \underset{k\in [t-K, t]}{\max }(y_r(k))\right) \end{aligned}$$
  where *K* refers to the maximum transport time in the water network, and $y_r$ refers to the chlorine concentration at the injection location over time—note that this generalizes for multiple injection sources.
- **The Mean-absolute-error (MAE)** as a standardized and easy-to-interpret error metrics:
  7 $$\begin{aligned} \frac{1}{T}\sum _{t=1}^{T} \left| {\hat{y}}_i(t) - y_i(t)\right| \end{aligned}$$
  where *T* refers to the length of the time horizon—i.e. length of the simulated scenario.
- **The running MAE** for evaluating the performance over time by accumulating the performance up to some given time point. By this, we evaluate whether the performance remains stable and robust throughout the entire duration. The running MAE is a function that maps a time horizon $k\le T$ to accumulated performance (i.e. MAE):
  8 $$\begin{aligned} f(k) = \frac{1}{k}\sum _{t=1}^{k} \left| {\hat{y}}_i(t) - y_i(t)\right| \end{aligned}$$
- **The amount of chlorine concentration which the model is over/underestimating**—i.e. indicating a bias for over or undershooting the true concentration. For this purpose, we sum up all positive and negative errors, and take their difference. A result close to zero indicates no bias, whereas a positive/negative result indicates a bias for over/underestimating the true concentration:
  9 $$\begin{aligned} \left( \sum _{t=1}^T \max (0, e_i(t))\right) - \left( \sum _{t=1}^T \max (0, -1 \cdot e_i(t))\right) \end{aligned}$$
  where
  10 $$\begin{aligned} e_i(t) = {\hat{y}}_i(t) - y_i(t) \end{aligned}$$

In addition to averaging scores across all nodes, we recommend comparing scores for each node individually. This approach allows us to assess whether errors and performance are uniformly distributed throughout the WDN or if certain nodes pose greater challenges than others. For example, a method’s performance might be influenced by the node’s distance from the reservoir (chlorine injection point)— i.e. predicting concentrations at nodes located farther away could be more challenging than at nodes closer to the injection site. One could also group nodes according to their transport delay—i.e. the time (e.g. time steps) it takes for a substance to travel from the injection point to the node of interest. Note that the transport delay might differ from the spatial distance (e.g. shortest path).

*Usage in Python* All the previously mentioned evaluation metrics are already implemented within the Python module included in the provided benchmark.

### Baseline Neural Surrogate Models

Here, we consider the problem of predicting chlorine states at all nodes in the network based on the chlorine injection at the reservoir and the flows at all pipes. This problem is equivalent to the simulation of the chlorine dynamics based on some given hydraulic simulation results like those performed in EPANET [34]. However, we aim for a more efficient and differentiable solution through surrogate models. Consequently, we introduce two baseline models for developing such a surrogate model within our proposed benchmark. Besides the dynamics such as mixing and decay, the major challenge in this context is accounting for transport delays—i.e. the time it takes for chlorine to reach specific nodes. This can be tackled using models with memory components, such as recurrent neural networks, or by employing models that locally integrate dynamics, like graph neural networks, which inherently create a memory component. We therefore implement and evaluate a recurrent neural network, and graph neural networks as baselines for chlorine state estimation. These neural networks can serve as state-of-the-art baselines for future research in machine learning-based chlorine state estimation. These models serve as examples of a *physics-guided* neural network and a *physics-encoded* neural network, respectively. These terms are part of a taxonomy proposed in [36] that describes models that derive physical relations purely from the data as *physics-guided* and models that directly adopt the structure of a known physical formula into the architecture as *physics-encoded*.

The Python implementation of the experiments is also included in the GitHub Repository of the benchmark^3^.
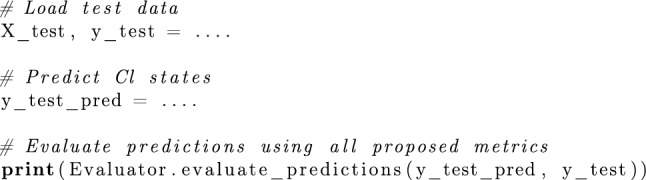


#### Recurrent Neural Network for Single Node Chlorine State Estimation

Our proposed deep recurrent neural network (RNN) for estimating chlorine concentration at a single node features two hidden Long Short-Term Memory (LSTM) [13] layers, with 128 and 64 neurons respectively, using tanh activation functions. A final dense layer with ReLU activation ensures non-negative predictions, as concentrations cannot be negative. The RNN takes as input the flow rate over time for every link in the WDN, as well as the chlorine concentration over time at the reservoir (injection point). Its task is to predict the chlorine concentration over time at a specified node in the WDN. The RNN is trained using the Adam optimizer [37] for up to 500 epochs, aiming to minimize the mean-squared error. Early stopping is implemented, halting the training if no improvement is observed over ten consecutive epochs. The dimensions of the hidden layers were optimized by a grid search (1–4 hidden layers, dimensions in [32, 64, 128, 256]) with a preference for lower values on the Net1 scenarios only, and recycled for all other networks due to the computational limitations of running a grid search for the larger water networks. Inputs to the RNN are standardized to prevent numerical issues during training. More details can be found in the accompanied Python code.

Because the data in our proposed benchmark data set (Sect. 3.1) is carefully crafted to model different dynamics such as transport delay, as well as breaking any correlations by random demand patterns and random chlorine injections, any model that is trained on it becomes a *physics-guided* model. Consequently, we refer to our proposed RNN-based surrogate model as a *physics-guided surrogate model.*

#### Graph Neural Network for Chlorine State Estimation

We now detail our GNN model for chlorine state estimation. We employ a message-passing neural network (MPNN) [17] to act as a PDE function and to model the dynamical system of chlorine levels. To do this, we first define the problem as an initial value problem with boundary conditions. The boundary conditions are chlorine injections at the reservoir and the initial state ${\textbf{w}}(0)$ is the first sample of a time series of the chlorine concentration data. The chlorine injections constitute a Dirichlet boundary condition $w_{\text {Reservoir}}(t) = w_{\text {Injection}}(t)$, because the chlorine concentration at the reservoir node is known. As described in Sect. 2.1, the governing equations in water quality are the advection and reaction PDEs. These PDEs are typically solved at a much smaller time scale and a high-resolution domain (the pipes of the WDN are subdivided into small segments). Instead of learning these known PDEs, we aim to approximate the unknown dynamical system that generates our measured data directly. I.e. we model chlorine distributions on the WDN graph at a coarser temporal resolution directly. This raises the issue that the chlorine concentrations ${\textbf{w}}(t)$ at time *t* do not fully determine $\frac{\partial {\textbf{w}}(t)}{\partial t}$, as chlorine distributed along the pipes is not contained in ${\textbf{w}}$. We address this by learning the PDE in a *d*-dimensional latent space ${\textbf{h}} \in {\mathbb {R}}^{N \times d}$, similar to the hidden state of an RNN. Consequently, we first project the initial condition ${\textbf{w}}(0)$ into the latent space, apply the GNN to integrate the initial state using Euler integration for *T* time steps and finally project the resulting outputs $H = [ {\textbf{h}}(0), .., {\textbf{h}}(T) ]$ back into the data space.

11 $$\begin{aligned} {\textbf{h}}(0)&= h_\phi ({\textbf{w}}(0)) \end{aligned}$$

12 $$\begin{aligned} {\textbf{h}}(t + \delta t)&= {\textbf{h}}(t) + \delta t \cdot f_\theta ({\textbf{h}}(t), {\textbf{q}}(t)) \end{aligned}$$

13 $$\begin{aligned} {\textbf{w}}(t)&= k_\psi ({\textbf{h}}(t)) \end{aligned}$$

where *h* and *k* are linear layers, *f* is a GNN that models the temporal change of chlorine concentrations, ${\textbf{q}}(t) \in {\mathbb {R}}^{|{\mathcal {E}}| \times 2}$ are normalized flow velocities and pipe lengths that we use as edge features, and $\delta t$ is the step size used for the Euler integration steps, we use $\delta t = \frac{1}{3}$ which corresponds to 10 minutes. Model parameters are denoted by $\phi$, $\theta$ and $\psi$. The GNN models

$$\begin{aligned} f_\theta ({\textbf{h}}(t), {\textbf{q}}(t)) = \frac{\partial {\textbf{h}}(t)}{\partial t} \end{aligned}$$

and computes residuals for a node *i* following the general formula of a GNN layer as given in Sect. 2.3 by

14 $$\begin{aligned} \frac{\partial {\textbf{h}}_i(t)}{\partial t} = \alpha \left( \sum _{j \in {\mathcal {N}}(i)} \, \phi \left( ({\textbf{h}}_j(t) - {\textbf{h}}_i(t)) \parallel {\textbf{q}}_{j,i}\right) - {\textbf{h}}_i(t) \right) , \end{aligned}$$

where $\alpha$ is a learnable scalar, $\phi$ is a ReLU-activated 2-layer MLP and $\cdot \parallel \cdot$ denotes vector concatenation. Note that because the GNN incorporates message-passing, spatial relationships can be modeled allowing $f_\theta$ to function as a PDE. The GNN is trained on sequences of length 30 time steps as we experienced stability problems when using longer training sequences. We use the Adam optimizer for 300 epochs minimizing the mean absolute error using a batch size of 32. We apply early stopping with a patience of 15 epochs and a learning rate schedule that reduces the learning rate by a factor of 0.5 when the validation loss does not decrease over 12 epochs. The chlorine values are normalized between 0 and 1, i.e. we divide by the maximal chlorine concentration observed at the injection nodes in the training split. 

### Experiments

#### Setup

We consider five different training configurations:

- Scenarios with normal demand patterns:
  - Spike chlorine injection patterns
  - Spike and random chlorine injection patterns
- Scenarios with randomized demand patterns:
  - Spike chlorine injection patterns
  - Spike and random chlorine injection patterns
- Scenarios with normal and randomized demand patterns: Spike and random chlorine injection patterns.

In every training setup, we randomly divide the scenarios into training (70%), validation (10%), and testing (20%) groups. If a configuration, such as a wave injection pattern, is not included in the training setup, we utilize all the scenarios of that configuration for evaluation.

We assess the performance of each injection pattern separately under both normal and randomized demand conditions using the proposed evaluation metrics (refer to “Evaluation Metrics”). A selection of the results (on the test set) is presented in “Analysis of Results”, with additional details provided in Appendix A. Our focus is on the “interesting” cases where our proposed baseline neural surrogate models do not perform well, highlighting challenging scenarios within the benchmark.

One such challenge is the presence of a tank in the *CY-DBP* network, separating it into two sub-networks (s. Figure 2). Tanks exhibit varying dynamics depending on their water level and can drastically smooth out the incoming signal. This prevents the application of a simple PDE-inspired GNN, as the local function that is learned by such a model cannot handle the discontinuity that the tank introduces. For this reason, we only benchmark the RNN on *CY-DBP* and leave the application of a GNN for future work.

*Computational Resources* All computations were performed on a GPU cluster whereby a single GPU (NVIDIA A40) was used for training a model. In the case of the RNNs (i.e. single node prediction), the training took (depending on early stopping) up to approx. 2 h on the CY-DBP network, and approx. 40 min in the case of Net1. All together, thanks to the cluster parallelization, training and evaluation of all RNN baselines runs within approx. 48 hours. While the entire benchmark is approx. 77.5 GB large, only a subset of those is required when training/evaluating a particular baseline model. For training an RNN baseline, the memory requirements are very modest. For instance, in the case of the CY-DBP network, a maximum of 350 MB on the GPU memory is required when training an RNN baseline for single-node prediction. The GNN has a slightly larger GPU memory requirement, as training is performed not on individual nodes but on the whole network. Nevertheless, training of the GNN on Net1 also takes approx. 40 min, this is mainly because it is trained on the first 30 time steps of each scenario and thus uses less data during training.

#### Analysis of Results

**Table 1:** Performance (*MAE*) on the test set of differently trained models—we report the average (over all nodes and scenarios) and standard deviation rounded to two decimal places

	Demands		Evaluation					
			Normal			Rand		
		Cl pattern(s)	Spike	Wave	Rand	Spike	Wave	Rand
Training	Normal	Spike	$0.01 {\scriptstyle \,\pm \,0.01 }$	$0.25 {\scriptstyle \,\pm \,0.09 }$	$0.22 {\scriptstyle \,\pm \,0.07 }$	$0.01 {\scriptstyle \,\pm \,0.01 }$	$0.23 {\scriptstyle \,\pm \,0.09 }$	$0.2 {\scriptstyle \,\pm \,0.07 }$
		Spike + Rand	$0.01 {\scriptstyle \,\pm \,0.01 }$	$0.11 {\scriptstyle \,\pm \,0.05 }$	$0.11 {\scriptstyle \,\pm \,0.04 }$	$0.01 {\scriptstyle \,\pm \,0.01 }$	$0.12 {\scriptstyle \,\pm \,0.05 }$	$0.12 {\scriptstyle \,\pm \,0.04 }$
	Rand	Spike	$0.02 {\scriptstyle \,\pm \,0.01 }$	$0.24 {\scriptstyle \,\pm \,0.09 }$	$0.24 {\scriptstyle \,\pm \,0.08 }$	$0.01 {\scriptstyle \,\pm \,0.01 }$	$0.22 {\scriptstyle \,\pm \,0.1 }$	$0.2 {\scriptstyle \,\pm \,0.09 }$
		Spike + Rand	$0.03 {\scriptstyle \,\pm \,0.02 }$	$0.16 {\scriptstyle \,\pm \,0.05 }$	$0.14 {\scriptstyle \,\pm \,0.04 }$	$0.01 {\scriptstyle \,\pm \,0.01 }$	$0.12 {\scriptstyle \,\pm \,0.04 }$	$0.11 {\scriptstyle \,\pm \,0.04 }$
	All	Spike + Rand	$0.01 {\scriptstyle \,\pm \,0.0 }$	$0.11 {\scriptstyle \,\pm \,0.04 }$	$0.1 {\scriptstyle \,\pm \,0.03 }$	$0.01 {\scriptstyle \,\pm \,0.0 }$	$0.11 {\scriptstyle \,\pm \,0.04 }$	$0.1 {\scriptstyle \,\pm \,0.04 }$

**Table 2:** Performance (*MAE*) on the test set of differently trained models—we report the average (over all nodes and scenarios) and standard deviation rounded to two decimal places

	Demands		Evaluation					
			Normal			Rand		
		Cl pattern(s)	Spike	Wave	Rand	Spike	Wave	Rand
Training	Normal	Spike	$0.05 {\scriptstyle \,\pm \,0.01}$	$0.39 {\scriptstyle \,\pm \,0.22}$	$0.41 {\scriptstyle \,\pm \,0.04}$	$0.06 {\scriptstyle \,\pm \,0.01}$	$0.41 {\scriptstyle \,\pm \,0.22}$	$0.42 {\scriptstyle \,\pm \,0.04}$
		Spike + Rand	$0.06 {\scriptstyle \,\pm \,0.04}$	$0.18 {\scriptstyle \,\pm \,0.13}$	$0.17 {\scriptstyle \,\pm \,0.05}$	$0.06 {\scriptstyle \,\pm \,0.05}$	$0.16 {\scriptstyle \,\pm \,0.13}$	$0.15 {\scriptstyle \,\pm \,0.07}$
	Rand	Spike	$0.05 {\scriptstyle \,\pm \,0.01}$	$0.4 {\scriptstyle \,\pm \,0.22}$	$0.43 {\scriptstyle \,\pm \,0.03}$	$0.05 {\scriptstyle \,\pm \,0.01}$	$0.42 {\scriptstyle \,\pm \,0.22}$	$0.44 {\scriptstyle \,\pm \,0.02}$
		Spike + Rand	$0.03 {\scriptstyle \,\pm \,0.01}$	$0.16 {\scriptstyle \,\pm \,0.13}$	$0.15 {\scriptstyle \,\pm \,0.05}$	$0.02 {\scriptstyle \,\pm \,0.01}$	$0.13 {\scriptstyle \,\pm \,0.12}$	$0.11 {\scriptstyle \,\pm \,0.06}$
	All	Spike + Rand	$0.04 {\scriptstyle \,\pm \,0.03}$	$0.16 {\scriptstyle \,\pm \,0.12}$	$0.15 {\scriptstyle \,\pm \,0.05}$	$0.04 {\scriptstyle \,\pm \,0.04}$	$0.13 {\scriptstyle \,\pm \,0.12}$	$0.11 {\scriptstyle \,\pm \,0.06}$

*General* Tables 1 and 2 show the performance (MAE Eq. (7) of the RNN and the GNN baseline surrogate models respectively. It can be observed that the RNN generally performs better than the GNN. However, while comparing these two surrogate models one should keep in mind that the RNN predicts single nodes only as we have separate models for each node. Instead, the GNN predicts all nodes simultaneously.

Comparing performances between different training configurations shows that the RNN is not as affected by changing from normal to randomized demand patterns as the GNN is. However, the results indicate that this is not an issue of generalization as both models show consistent transfer learning capabilities w.r.t. the demand pattern. Finally, including random chlorine injection patterns in the training generally shows a positive influence. Models trained on random injection patterns also show the best overall performance, especially for the GNN model. We include evaluations on the Net1 network and tables showing the other metrics in the Appendix (Tables 3, 4, 5, 6, 7, 8, 9 and 10), which support these findings.

*Physically plausible concentration estimates* Recall that our proposed benchmark comes with two metrics checking for severe violations of physics—i.e. non-negativity of concentrations and an upper bound based on the transport delay.

Note that by design both RNN and GNN can not output negative numbers—i.e. fulfilling the non-negativity constraint of concentrations. When it comes to the upper bound (based on the transport delay) as proposed in Eq. (6, we observe (see Tables 4, 9, 15) that the RNN almost always satisfies this upper bound, predicting physically plausible chlorine concentrations. This is quite remarkable given the fact that it does not contain any explicit physics constraints. All the physics constraints are implicitly in the carefully created training data, demonstrating the benefit of this *physics-guided* approach. We the exception of the spike injection pattern, the same observation can be made for the GNN (see Tables 6, 12)

*Bias for over or underestimating* From the results (Tables 3, 8, 14) we see that almost always the RNN has a bias towards underestimating the true chlorine concentration if it makes an error. This is a relevant finding since such a bias (positive or negative) might propagate in downstream tasks such as control. We also see that the GNN (Tables 5, 11) shows an even greater tendency to underestimate chlorine. To this end, both models show significantly lower bias when random injection patterns are included in the training set.

**Fig. 4:**
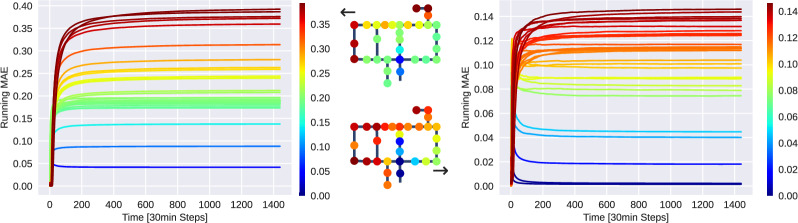
Averaged running MAE of the *RNN* on the *Hanoi* network for randomized injections patterns and random demands (test set)—each colored line represents a different node. *Left:* Training was conducted using spike injection patterns. *Right:* Training involved both spike and random injection patterns. In both settings, the training was performed on randomized demand patterns. The colors show the total (average) MAE per node (last time step of the running MAE)

**Fig. 5:**
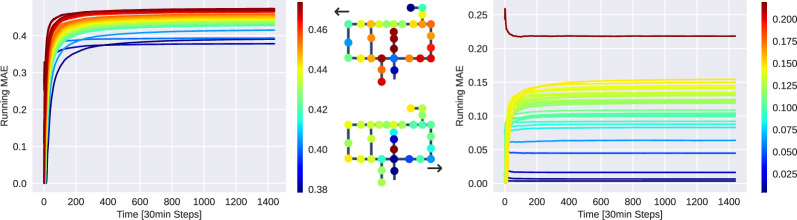
Averaged running MAE of the GNN on the Hanoi network for randomized injections patterns and random demands (test set)—each colored line represents a different node. *Left:* Training was conducted using spike injection patterns. *Right:* Training involved both spike and random injection patterns. In both settings, the training was performed on randomized demand patterns. The colors show the total (average) MAE per node (last time step of the running MAE)

**Fig. 6:**
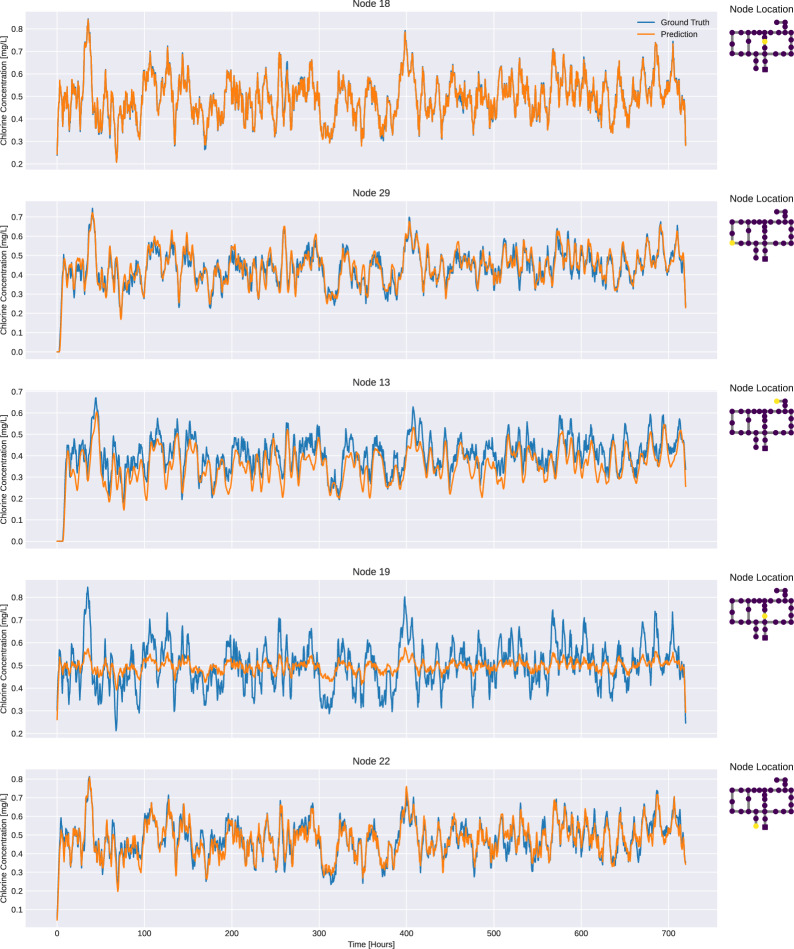
GNN chlorine predictions vs. ground truth of different nodes in the Hanoi network. The GNN was trained on all demand patterns and *spike+random* injection patterns. Visualized are results on the test set with *random* demands and the *random* injection pattern

*Performance over time and space* In Fig. 4, we show the running MAE Eq. (8) of the RNN on the Hanoi network. Our observations indicate that: (1) The performance remains stable over time. (2) Training on randomized injection patterns considerably enhances performance. (3) The RNN’s performance can vary significantly across different nodes. Further analysis shows that nodes located farther from the chlorine injection point at the reservoir (indicated by the node with zero error in dark blue) present more challenges than nodes closer to the injection point. Similar performance variation can be observed in the GNN too, as indicated by Figs. 5 and 6, although, for the GNN, the performance does not seem to depend as much on the distance to the injection node. The observed variability can be attributed to several key factors. First, as chlorine travels through the network, it undergoes reaction processes that lead to gradual decay. Nodes farther from the injection point experience longer travel times, allowing for extended reaction durations, which increases uncertainty in concentration predictions. Second, it is well established in the scientific literature [6, 38] that variability in water flow is the greatest source of uncertainty in water quality models. The flow paths to distant nodes are often more complex, involving varying pipe diameters and multiple junctions where water mixing occurs. These factors introduce non-linearities in chlorine transport, making it more difficult for machine learning models to generalize.

To improve performance at challenging nodes, some alternative strategies can be explored, such as generating additional training data specifically targeting long-distance chlorine transport scenarios. Additionally, adjusting model loss functions to place greater emphasis on performance at distant nodes could help capture long-range dependencies more effectively.

## Conclusion and Summary

In this work, we introduced a benchmark for chlorine state estimation in water distribution networks, featuring various chlorine injection patterns to capture diverse physical dynamics. This benchmark includes a comprehensive dataset comprising 18,000 scenarios and is supported by a Python module to facilitate easy access to the data and evaluation metrics within Python. Additionally, we introduce neural surrogate baselines using two different (physics-guided/informed) deep neural networks for chlorine state estimation and assess their performance using the proposed benchmark.

The empirical evaluation reveals that the RNN-based surrogate model typically outperforms the GNN-based surrogate model. When examining various training configurations, it appears that the RNN is less impacted by transitioning from standard to randomized demand patterns compared to the GNN. Nevertheless, the findings suggest that this is not a matter of generalization, as both models demonstrate consistent performance with respect to the demand pattern used in the evaluation dataset. Additionally, incorporating random chlorine injection patterns during training generally has a positive impact, with models trained on these patterns achieving the best overall performance. In terms of long-term robustness, both the RNN and GNN exhibit stable performance over time. However, there is a noticeable variation in performance across nodes, which depends significantly on their distance from the chlorine injection point. This suggests that substantial distances between the chlorine injection point and the target node present a significant challenge for the neural networks (also likely due to the longer time horizon).

*Future work* The proposed benchmark, along with the two baseline neural surrogate models, lays a foundation for future research in machine learning-based chlorine state estimation. In order to align with real-world constraints, future studies may move beyond the assumption of complete knowledge of all variables and explore configurations involving flow and chlorine sensors as well as uncertainties such as noisy measurements. Another interesting and highly important direction for future research would be to incorporate advanced reactions, such as additional species like contaminants, as well as time-varying and uncertain reactions.

Building on the presented baseline models, a promising direction for future research is refining the current PDE-inspired GNN. Our model is based on advection dynamics, which may not fully align with the partial observability of chlorine and the coarse temporal resolution of the proposed datasets. This mismatch adds complexity to the learning task and could be mitigated by further tailoring the PDE-based model to better account for these characteristics. Furthermore, other integration methods like Runge-Kutta, or implicit schemes could further improve the results.

## Data Availability

All data is publicly available.

## Appendix A. Additional Empirical Results

### Appendix A.1 Hanoi

See Tables 3, 4, 5, 6, and Figs. 5, 6

**Table 3:** Performance (over/underestimation Eq. (9) on the test set of differently trained models—we report the average (over all nodes and scenarios) and standard deviation rounded to two decimal places

	Demands		Evaluation					
			Normal			Rand		
		Cl pattern(s)	Spike	Wave	Rand	Spike	Wave	Rand
Training	Normal	Spike	$-3.42 {\scriptstyle \,\pm \,12.49 }$	$-208.14 {\scriptstyle \,\pm \,188.2 }$	$-72.82 {\scriptstyle \,\pm \,187.42 }$	$-3.71 {\scriptstyle \,\pm \,13.35 }$	$-186.38 {\scriptstyle \,\pm \,179.13 }$	$-29.91 {\scriptstyle \,\pm \,196.04 }$
		Spike + Rand	$-0.44 {\scriptstyle \,\pm \,1.01 }$	$-59.82 {\scriptstyle \,\pm \,23.11 }$	$2.39 {\scriptstyle \,\pm \,6.61 }$	$0.58 {\scriptstyle \,\pm \,3.2 }$	$-65.78 {\scriptstyle \,\pm \,24.66 }$	$-4.51 {\scriptstyle \,\pm \,9.11 }$
	Rand	Spike	$-11.93 {\scriptstyle \,\pm \,14.54 }$	$-280.17 {\scriptstyle \,\pm \,159.83 }$	$-220.21 {\scriptstyle \,\pm \,152.74 }$	$-1.05 {\scriptstyle \,\pm \,1.07 }$	$-276.6 {\scriptstyle \,\pm \,165.7 }$	$-201.04 {\scriptstyle \,\pm \,179.97 }$
		Spike + Rand	$18.81 {\scriptstyle \,\pm \,24.98 }$	$-70.54 {\scriptstyle \,\pm \,31.83 }$	$-14.47 {\scriptstyle \,\pm \,28.21 }$	$-0.37 {\scriptstyle \,\pm \,1.01 }$	$-64.96 {\scriptstyle \,\pm \,25.14 }$	$0.01 {\scriptstyle \,\pm \,6.26 }$
	All	Spike + Rand	$-0.77 {\scriptstyle \,\pm \,1.04 }$	$-65.81 {\scriptstyle \,\pm \,23.69 }$	$-2.04 {\scriptstyle \,\pm \,7.75 }$	$-0.43 {\scriptstyle \,\pm \,1.05 }$	$-66.74 {\scriptstyle \,\pm \,26.3 }$	$-0.66 {\scriptstyle \,\pm \,8.52 }$

**Table 4:** Performance (Satisfaction of an upper bound satisfaction based on transport delay Eq. (6) on the test set of differently trained models—we report the average (over all nodes and scenarios) and standard deviation rounded to two decimal places

	Demands		Evaluation					
			Normal			Rand		
		Cl pattern(s)	Spike	Wave	Rand	Spike	Wave	Rand
Training	Normal	Spike	$1.0 {\scriptstyle \,\pm \,0.01 }$	$0.98 {\scriptstyle \,\pm \,0.02 }$	$1.0 {\scriptstyle \,\pm \,0.01 }$	$0.99 {\scriptstyle \,\pm \,0.01 }$	$0.98 {\scriptstyle \,\pm \,0.02 }$	$1.0 {\scriptstyle \,\pm \,0.0 }$
		Spike + Rand	$0.99 {\scriptstyle \,\pm \,0.01 }$	$1.0 {\scriptstyle \,\pm \,0.01 }$	$1.0 {\scriptstyle \,\pm \,0.01 }$	$0.98 {\scriptstyle \,\pm \,0.04 }$	$0.99 {\scriptstyle \,\pm \,0.01 }$	$1.0 {\scriptstyle \,\pm \,0.0 }$
	Rand	Spike	$0.97 {\scriptstyle \,\pm \,0.04 }$	$0.98 {\scriptstyle \,\pm \,0.01 }$	$1.0 {\scriptstyle \,\pm \,0.0 }$	$1.0 {\scriptstyle \,\pm \,0.01 }$	$0.99 {\scriptstyle \,\pm \,0.01 }$	$1.0 {\scriptstyle \,\pm \,0.0 }$
		Spike + Rand	$0.9 {\scriptstyle \,\pm \,0.06 }$	$0.97 {\scriptstyle \,\pm \,0.02 }$	$1.0 {\scriptstyle \,\pm \,0.01 }$	$0.99 {\scriptstyle \,\pm \,0.01 }$	$1.0 {\scriptstyle \,\pm \,0.01 }$	$1.0 {\scriptstyle \,\pm \,0.01 }$
	All	Spike + Rand	$1.0 {\scriptstyle \,\pm \,0.01 }$	$1.0 {\scriptstyle \,\pm \,0.01 }$	$1.0 {\scriptstyle \,\pm \,0.01 }$	$0.99 {\scriptstyle \,\pm \,0.01 }$	$1.0 {\scriptstyle \,\pm \,0.01 }$	$1.0 {\scriptstyle \,\pm \,0.01 }$

**Table 5:** Performance (Over/underestimation) on the test set of differently trained models—we report the average (over all nodes and scenarios) and standard deviation rounded to two decimal places

	Demands		Evaluation					
			Normal			Rand		
		Cl pattern(s)	Spike	Wave	Rand	Spike	Wave	Rand
Training	Normal	Spike	$-51.13 {\scriptstyle \,\pm \,13.69}$	$-482.43 {\scriptstyle \,\pm \,312.18}$	$-579.1 {\scriptstyle \,\pm \,78.7}$	$-57.35 {\scriptstyle \,\pm \,12.3}$	$-532.44 {\scriptstyle \,\pm \,319.28}$	$-598.18 {\scriptstyle \,\pm \,79.58}$
		Spike + Rand	$37.14 {\scriptstyle \,\pm \,69.83}$	$-123.72 {\scriptstyle \,\pm \,154.99}$	$-81.62 {\scriptstyle \,\pm \,93.23}$	$45.05 {\scriptstyle \,\pm \,78.16}$	$-119.45 {\scriptstyle \,\pm \,142.48}$	$-55.2 {\scriptstyle \,\pm \,81.9}$
	Rand	Spike	$-68.15 {\scriptstyle \,\pm \,12.75}$	$-556.26 {\scriptstyle \,\pm \,307.07}$	$-616.86 {\scriptstyle \,\pm \,44.1}$	$-72.05 {\scriptstyle \,\pm \,10.86}$	$-591.8 {\scriptstyle \,\pm \,308.55}$	$-635.05 {\scriptstyle \,\pm \,38.85}$
		Spike + Rand	$-8.68 {\scriptstyle \,\pm \,10.23}$	$-111.61 {\scriptstyle \,\pm \,140.63}$	$-22.75 {\scriptstyle \,\pm \,43.13}$	$-9.58 {\scriptstyle \,\pm \,7.16}$	$-111.7 {\scriptstyle \,\pm \,135.03}$	$-10.14 {\scriptstyle \,\pm \,18.51}$
	All	Spike + Rand	$31.03 {\scriptstyle \,\pm \,46.84}$	$-108.83 {\scriptstyle \,\pm \,153.45}$	$-31.23 {\scriptstyle \,\pm \,52.67}$	$32.65 {\scriptstyle \,\pm \,47.16}$	$-95.62 {\scriptstyle \,\pm \,144.05}$	$-3.54 {\scriptstyle \,\pm \,19.16}$

**Table 6:** Performance (Satisfaction of an upper bound based on transport delay Eq. (6) on the test set of differently trained models – we report the average (over all nodes and scenarios) and standard deviation rounded to two decimal places

	Demands		Evaluation					
			Normal			Rand		
		Cl pattern(s)	Spike	Wave	Rand	Spike	Wave	Rand
Training	Normal	Spike	$0.69 {\scriptstyle \,\pm \,0.46}$	$1.0 {\scriptstyle \,\pm \,0.06}$	$1.0 {\scriptstyle \,\pm \,0.0}$	$0.73 {\scriptstyle \,\pm \,0.44}$	$1.0 {\scriptstyle \,\pm \,0.02}$	$1.0 {\scriptstyle \,\pm \,0.0}$
		Spike + Rand	$0.56 {\scriptstyle \,\pm \,0.5}$	$0.95 {\scriptstyle \,\pm \,0.22}$	$1.0 {\scriptstyle \,\pm \,0.05}$	$0.54 {\scriptstyle \,\pm \,0.5}$	$0.98 {\scriptstyle \,\pm \,0.13}$	$1.0 {\scriptstyle \,\pm \,0.06}$
	Rand	Spike	$0.71 {\scriptstyle \,\pm \,0.45}$	$1.0 {\scriptstyle \,\pm \,0.06}$	$1.0 {\scriptstyle \,\pm \,0.0}$	$0.73 {\scriptstyle \,\pm \,0.45}$	$1.0 {\scriptstyle \,\pm \,0.02}$	$1.0 {\scriptstyle \,\pm \,0.0}$
		Spike + Rand	$0.71 {\scriptstyle \,\pm \,0.45}$	$0.98 {\scriptstyle \,\pm \,0.14}$	$1.0 {\scriptstyle \,\pm \,0.05}$	$0.73 {\scriptstyle \,\pm \,0.44}$	$0.99 {\scriptstyle \,\pm \,0.08}$	$1.0 {\scriptstyle \,\pm \,0.03}$
	All	Spike + Rand	$0.56 {\scriptstyle \,\pm \,0.5}$	$0.96 {\scriptstyle \,\pm \,0.21}$	$1.0 {\scriptstyle \,\pm \,0.05}$	$0.53 {\scriptstyle \,\pm \,0.5}$	$0.97 {\scriptstyle \,\pm \,0.16}$	$1.0 {\scriptstyle \,\pm \,0.03}$

### Appendix A.2. Net1

See Tables 7, 8, 9, 10, 11 and 12.

**Table 7:** Performance (MAE) on the test set of differently trained models—we report the average (over all nodes and scenarios) and standard deviation rounded to two decimal places

	Demands		Evaluation					
			Normal			Rand		
		Cl pattern(s)	Spike	Wave	Rand	Spike	Wave	Rand
Training	Normal	Spike	$0.02 {\scriptstyle \,\pm \,0.01 }$	$0.1 {\scriptstyle \,\pm \,0.04 }$	$0.09 {\scriptstyle \,\pm \,0.04 }$	$0.02 {\scriptstyle \,\pm \,0.01 }$	$0.1 {\scriptstyle \,\pm \,0.04 }$	$0.1 {\scriptstyle \,\pm \,0.05 }$
		Spike + Rand	$0.02 {\scriptstyle \,\pm \,0.01 }$	$0.08 {\scriptstyle \,\pm \,0.03 }$	$0.08 {\scriptstyle \,\pm \,0.04 }$	$0.02 {\scriptstyle \,\pm \,0.01 }$	$0.08 {\scriptstyle \,\pm \,0.03 }$	$0.08 {\scriptstyle \,\pm \,0.04 }$
	Rand	Spike	$0.02 {\scriptstyle \,\pm \,0.01 }$	$0.11 {\scriptstyle \,\pm \,0.04 }$	$0.1 {\scriptstyle \,\pm \,0.04 }$	$0.02 {\scriptstyle \,\pm \,0.01 }$	$0.1 {\scriptstyle \,\pm \,0.04 }$	$0.1 {\scriptstyle \,\pm \,0.05 }$
		Spike + Rand	$0.02 {\scriptstyle \,\pm \,0.01 }$	$0.08 {\scriptstyle \,\pm \,0.03 }$	$0.08 {\scriptstyle \,\pm \,0.04 }$	$0.02 {\scriptstyle \,\pm \,0.01 }$	$0.08 {\scriptstyle \,\pm \,0.03 }$	$0.08 {\scriptstyle \,\pm \,0.04 }$
	All	Spike + Rand	$0.02 {\scriptstyle \,\pm \,0.01 }$	$0.06 {\scriptstyle \,\pm \,0.03 }$	$0.07 {\scriptstyle \,\pm \,0.03 }$	$0.02 {\scriptstyle \,\pm \,0.01 }$	$0.06 {\scriptstyle \,\pm \,0.03 }$	$0.08 {\scriptstyle \,\pm \,0.04 }$

**Table 8:** Performance (over/underestimation) on the test set of differently trained models—we report the average (over all nodes and scenarios) and standard deviation rounded to two decimal places

	Demands		Evaluation					
			Normal			Rand		
		Cl pattern(s)	Spike	Wave	Rand	Spike	Wave	Rand
Training	Normal	Spike	$-1.22 {\scriptstyle \,\pm \,1.4 }$	$-17.57 {\scriptstyle \,\pm \,21.1 }$	$-2.38 {\scriptstyle \,\pm \,11.46 }$	$-2.25 {\scriptstyle \,\pm \,3.11 }$	$-21.6 {\scriptstyle \,\pm \,24.79 }$	$-2.37 {\scriptstyle \,\pm \,15.61 }$
		Spike + Rand	$-0.99 {\scriptstyle \,\pm \,1.4 }$	$-19.3 {\scriptstyle \,\pm \,7.84 }$	$0.52 {\scriptstyle \,\pm \,3.09 }$	$1.77 {\scriptstyle \,\pm \,2.28 }$	$-19.65 {\scriptstyle \,\pm \,8.73 }$	$0.65 {\scriptstyle \,\pm \,4.14 }$
	Rand	Spike	$-1.2 {\scriptstyle \,\pm \,4.04 }$	$-21.04 {\scriptstyle \,\pm \,17.72 }$	$-5.17 {\scriptstyle \,\pm \,9.56 }$	$-0.76 {\scriptstyle \,\pm \,1.66 }$	$-19.56 {\scriptstyle \,\pm \,20.6 }$	$-1.16 {\scriptstyle \,\pm \,10.32 }$
		Spike + Rand	$0.15 {\scriptstyle \,\pm \,4.92 }$	$-21.16 {\scriptstyle \,\pm \,9.4 }$	$-0.53 {\scriptstyle \,\pm \,6.9 }$	$-2.5 {\scriptstyle \,\pm \,2.73 }$	$-21.4 {\scriptstyle \,\pm \,8.94 }$	$-0.43 {\scriptstyle \,\pm \,4.83 }$
	All	Spike + Rand	$-0.63 {\scriptstyle \,\pm \,2.54 }$	$-18.93 {\scriptstyle \,\pm \,7.73 }$	$-1.28 {\scriptstyle \,\pm \,4.16 }$	$-0.67 {\scriptstyle \,\pm \,2.44 }$	$-20.21 {\scriptstyle \,\pm \,7.73 }$	$-0.55 {\scriptstyle \,\pm \,4.64 }$

**Table 9:** Performance (satisfaction of an upper bound based on transport delay Eq. (6) on the test set of differently trained models – we report the average (over all nodes and scenarios) and standard deviation rounded to two decimal places

	Demands		Evaluation					
			Normal			Rand		
		Cl pattern(s)	Spike	Wave	Rand	Spike	Wave	Rand
Training	Normal	Spike	$1.0 {\scriptstyle \,\pm \,0.0 }$	$1.0 {\scriptstyle \,\pm \,0.0 }$	$1.0 {\scriptstyle \,\pm \,0.0 }$	$1.0 {\scriptstyle \,\pm \,0.01 }$	$0.99 {\scriptstyle \,\pm \,0.01 }$	$1.0 {\scriptstyle \,\pm \,0.0 }$
		Spike + Rand	$0.99 {\scriptstyle \,\pm \,0.01 }$	$1.0 {\scriptstyle \,\pm \,0.0 }$	$1.0 {\scriptstyle \,\pm \,0.0 }$	$1.0 {\scriptstyle \,\pm \,0.01 }$	$1.0 {\scriptstyle \,\pm \,0.01 }$	$1.0 {\scriptstyle \,\pm \,0.0 }$
	Rand	Spike	$0.99 {\scriptstyle \,\pm \,0.01 }$	$0.99 {\scriptstyle \,\pm \,0.01 }$	$1.0 {\scriptstyle \,\pm \,0.0 }$	$1.0 {\scriptstyle \,\pm \,0.01 }$	$0.99 {\scriptstyle \,\pm \,0.01 }$	$1.0 {\scriptstyle \,\pm \,0.0 }$
		Spike + Rand	$0.99 {\scriptstyle \,\pm \,0.01 }$	$0.99 {\scriptstyle \,\pm \,0.0 }$	$1.0 {\scriptstyle \,\pm \,0.0 }$	$1.0 {\scriptstyle \,\pm \,0.01 }$	$1.0 {\scriptstyle \,\pm \,0.0 }$	$1.0 {\scriptstyle \,\pm \,0.0 }$
	All	Spike + Rand	$0.99 {\scriptstyle \,\pm \,0.01 }$	$1.0 {\scriptstyle \,\pm \,0.0 }$	$1.0 {\scriptstyle \,\pm \,0.0 }$	$1.0 {\scriptstyle \,\pm \,0.01 }$	$1.0 {\scriptstyle \,\pm \,0.0 }$	$1.0 {\scriptstyle \,\pm \,0.0 }$

**Table 10:** Performance (MAE) on the test set of differently trained models—we report the average (over all nodes and scenarios) and standard deviation rounded to two decimal places

	Demands		Evaluation					
			Normal			Rand		
		Cl pattern(s)	Spike	Wave	Rand	Spike	Wave	Rand
Training	Normal	Spike	$0.12 {\scriptstyle \,\pm \,0.04}$	$0.19 {\scriptstyle \,\pm \,0.11}$	$0.19 {\scriptstyle \,\pm \,0.03}$	$0.13 {\scriptstyle \,\pm \,0.05}$	$0.22 {\scriptstyle \,\pm \,0.12}$	$0.19 {\scriptstyle \,\pm \,0.03}$
		Spike + Rand	$0.06 {\scriptstyle \,\pm \,0.03}$	$0.15 {\scriptstyle \,\pm \,0.11}$	$0.14 {\scriptstyle \,\pm \,0.06}$	$0.07 {\scriptstyle \,\pm \,0.04}$	$0.17 {\scriptstyle \,\pm \,0.12}$	$0.15 {\scriptstyle \,\pm \,0.06}$
	Rand	Spike	$0.08 {\scriptstyle \,\pm \,0.04}$	$0.18 {\scriptstyle \,\pm \,0.11}$	$0.19 {\scriptstyle \,\pm \,0.03}$	$0.09 {\scriptstyle \,\pm \,0.05}$	$0.2 {\scriptstyle \,\pm \,0.12}$	$0.19 {\scriptstyle \,\pm \,0.03}$
		Spike + Rand	$0.04 {\scriptstyle \,\pm \,0.02}$	$0.11 {\scriptstyle \,\pm \,0.08}$	$0.11 {\scriptstyle \,\pm \,0.04}$	$0.04 {\scriptstyle \,\pm \,0.03}$	$0.13 {\scriptstyle \,\pm \,0.09}$	$0.11 {\scriptstyle \,\pm \,0.05}$
	All	Spike + Rand	$0.04 {\scriptstyle \,\pm \,0.02}$	$0.11 {\scriptstyle \,\pm \,0.09}$	$0.1 {\scriptstyle \,\pm \,0.04}$	$0.04 {\scriptstyle \,\pm \,0.02}$	$0.13 {\scriptstyle \,\pm \,0.1}$	$0.1 {\scriptstyle \,\pm \,0.05}$

**Table 11:** Performance (over/underestimation) on the test set of differently trained models—we report the average (over all nodes and scenarios) and standard deviation rounded to two decimal places

	Demands		Evaluation					
			Normal			Rand		
		*Cl pattern(s)*	Spike	Wave	Rand	Spike	Wave	Rand
Training	Normal	Spike	$9.26 {\scriptstyle \,\pm \,80.03}$	$-141.5 {\scriptstyle \,\pm \,191.05}$	$-164.28 {\scriptstyle \,\pm \,94.04}$	$-9.68 {\scriptstyle \,\pm \,88.48}$	$-172.07 {\scriptstyle \,\pm \,205.97}$	$-169.11 {\scriptstyle \,\pm \,89.99}$
		Spike + Rand	$-29.0 {\scriptstyle \,\pm \,54.87}$	$-121.61 {\scriptstyle \,\pm \,161.23}$	$-95.97 {\scriptstyle \,\pm \,125.22}$	$-34.66 {\scriptstyle \,\pm \,62.42}$	$-144.21 {\scriptstyle \,\pm \,170.83}$	$-93.72 {\scriptstyle \,\pm \,122.57}$
	Rand	Spike	$-41.36 {\scriptstyle \,\pm \,64.9}$	$-147.55 {\scriptstyle \,\pm \,174.09}$	$-168.69 {\scriptstyle \,\pm \,95.54}$	$-50.23 {\scriptstyle \,\pm \,73.4}$	$-169.43 {\scriptstyle \,\pm \,180.91}$	$-161.38 {\scriptstyle \,\pm \,91.33}$
		Spike + Rand	$-13.67 {\scriptstyle \,\pm \,21.08}$	$-80.63 {\scriptstyle \,\pm \,81.92}$	$-53.67 {\scriptstyle \,\pm \,54.65}$	$-18.53 {\scriptstyle \,\pm \,30.75}$	$-103.81 {\scriptstyle \,\pm \,97.62}$	$-65.49 {\scriptstyle \,\pm \,67.9}$
	All	Spike + Rand	$-15.44 {\scriptstyle \,\pm \,20.1}$	$-100.65 {\scriptstyle \,\pm \,105.47}$	$-49.2 {\scriptstyle \,\pm \,39.23}$	$-20.05 {\scriptstyle \,\pm \,23.64}$	$-124.71 {\scriptstyle \,\pm \,114.77}$	$-51.79 {\scriptstyle \,\pm \,40.53}$

**Table 12:** Performance (satisfaction of an upper bound based on transport delay Eq. (6) on the test set of differently trained models—we report the average (over all nodes and scenarios) and standard deviation rounded to two decimal places

	Demands		Evaluation					
			Normal			Rand		
		Cl pattern(s)	Spike	Wave	Rand	Spike	Wave	Rand
Training	Normal	Spike	$0.98 {\scriptstyle \,\pm \,0.14}$	$0.94 {\scriptstyle \,\pm \,0.24}$	$1.0 {\scriptstyle \,\pm \,0.02}$	$0.99 {\scriptstyle \,\pm \,0.1}$	$0.95 {\scriptstyle \,\pm \,0.21}$	$1.0 {\scriptstyle \,\pm \,0.02}$
		Spike + Rand	$0.98 {\scriptstyle \,\pm \,0.14}$	$0.98 {\scriptstyle \,\pm \,0.15}$	$1.0 {\scriptstyle \,\pm \,0.07}$	$0.99 {\scriptstyle \,\pm \,0.12}$	$0.98 {\scriptstyle \,\pm \,0.13}$	$0.99 {\scriptstyle \,\pm \,0.08}$
	Rand	Spike	$0.98 {\scriptstyle \,\pm \,0.13}$	$0.97 {\scriptstyle \,\pm \,0.17}$	$1.0 {\scriptstyle \,\pm \,0.01}$	$0.99 {\scriptstyle \,\pm \,0.09}$	$0.98 {\scriptstyle \,\pm \,0.14}$	$1.0 {\scriptstyle \,\pm \,0.01}$
		Spike + Rand	$0.98 {\scriptstyle \,\pm \,0.14}$	$0.99 {\scriptstyle \,\pm \,0.11}$	$1.0 {\scriptstyle \,\pm \,0.02}$	$0.99 {\scriptstyle \,\pm \,0.12}$	$0.99 {\scriptstyle \,\pm \,0.08}$	$1.0 {\scriptstyle \,\pm \,0.02}$
	All	Spike + Rand	$0.98 {\scriptstyle \,\pm \,0.14}$	$0.99 {\scriptstyle \,\pm \,0.11}$	$1.0 {\scriptstyle \,\pm \,0.03}$	$0.99 {\scriptstyle \,\pm \,0.11}$	$0.99 {\scriptstyle \,\pm \,0.09}$	$1.0 {\scriptstyle \,\pm \,0.03}$

### Appendix A.3. CY-DBP

See Tables 13, 14 and 15.

**Table 13:** Performance (MAE) on the test set of differently trained models—we report the average (over all nodes and scenarios) and standard deviation rounded to two decimal places

	Demands		Evaluation					
			Normal			Rand		
		Cl pattern(s)	Spike	Wave	Rand	Spike	Wave	Rand
Training	Normal	Spike	$0.05 {\scriptstyle \,\pm \,0.01 }$	$0.37 {\scriptstyle \,\pm \,0.09 }$	$0.38 {\scriptstyle \,\pm \,0.09 }$	$0.05 {\scriptstyle \,\pm \,0.01 }$	$0.39 {\scriptstyle \,\pm \,0.1 }$	$0.39 {\scriptstyle \,\pm \,0.09 }$
		Spike + Rand	$0.02 {\scriptstyle \,\pm \,0.01 }$	$0.16 {\scriptstyle \,\pm \,0.1 }$	$0.07 {\scriptstyle \,\pm \,0.13 }$	$0.03 {\scriptstyle \,\pm \,0.03 }$	$0.18 {\scriptstyle \,\pm \,0.1 }$	$0.09 {\scriptstyle \,\pm \,0.13 }$
	Rand	Spike	$0.05 {\scriptstyle \,\pm \,0.01 }$	$0.37 {\scriptstyle \,\pm \,0.09 }$	$0.38 {\scriptstyle \,\pm \,0.09 }$	$0.05 {\scriptstyle \,\pm \,0.01 }$	$0.39 {\scriptstyle \,\pm \,0.1 }$	$0.39 {\scriptstyle \,\pm \,0.1 }$
		Spike + Rand	$0.02 {\scriptstyle \,\pm \,0.01 }$	$0.13 {\scriptstyle \,\pm \,0.06 }$	$0.05 {\scriptstyle \,\pm \,0.07 }$	$0.01 {\scriptstyle \,\pm \,0.01 }$	$0.14 {\scriptstyle \,\pm \,0.06 }$	$0.03 {\scriptstyle \,\pm \,0.07 }$
	All	Spike + Rand	$0.01 {\scriptstyle \,\pm \,0.01 }$	$0.14 {\scriptstyle \,\pm \,0.07 }$	$0.04 {\scriptstyle \,\pm \,0.09 }$	$0.01 {\scriptstyle \,\pm \,0.01 }$	$0.15 {\scriptstyle \,\pm \,0.07 }$	$0.04 {\scriptstyle \,\pm \,0.09 }$

**Table 14:** Performance (over/underestimation Eq. (9) on the test set of differently trained models—we report the average (over all nodes and scenarios) and standard deviation rounded to two decimal places

	Demands		Evaluation					
			Normal			Rand		
		Cl pattern(s)	Spike	Wave	Rand	Spike	Wave	Rand
Training	Normal	Spike	$-69.5 {\scriptstyle \,\pm \,20.0 }$	$-534.0 {\scriptstyle \,\pm \,133.5 }$	$-550.12 {\scriptstyle \,\pm \,139.9 }$	$-71.88 {\scriptstyle \,\pm \,19.46 }$	$-561.43 {\scriptstyle \,\pm \,139.02 }$	$-556.91 {\scriptstyle \,\pm \,140.05 }$
		Spike + Rand	$-10.62 {\scriptstyle \,\pm \,25.47 }$	$-161.38 {\scriptstyle \,\pm \,156.64 }$	$-83.06 {\scriptstyle \,\pm \,194.99 }$	$-3.22 {\scriptstyle \,\pm \,54.5 }$	$-191.62 {\scriptstyle \,\pm \,159.55 }$	$-98.82 {\scriptstyle \,\pm \,193.78 }$
	Rand	Spike	$-71.36 {\scriptstyle \,\pm \,17.71 }$	$-537.3 {\scriptstyle \,\pm \,131.16 }$	$-554.1 {\scriptstyle \,\pm \,135.27 }$	$-73.48 {\scriptstyle \,\pm \,17.83 }$	$-564.56 {\scriptstyle \,\pm \,137.97 }$	$-560.67 {\scriptstyle \,\pm \,137.01 }$
		Spike + Rand	$-13.24 {\scriptstyle \,\pm \,16.03 }$	$-138.96 {\scriptstyle \,\pm \,78.33 }$	$-44.2 {\scriptstyle \,\pm \,98.1 }$	$-2.96 {\scriptstyle \,\pm \,13.77 }$	$-125.11 {\scriptstyle \,\pm \,78.86 }$	$-21.56 {\scriptstyle \,\pm \,95.82 }$
	All	Spike + Rand	$-4.23 {\scriptstyle \,\pm \,16.37 }$	$-116.59 {\scriptstyle \,\pm \,100.51 }$	$-33.7 {\scriptstyle \,\pm \,124.12 }$	$-4.46 {\scriptstyle \,\pm \,16.65 }$	$-131.75 {\scriptstyle \,\pm \,103.61 }$	$-34.18 {\scriptstyle \,\pm \,125.53 }$

**Table 15:** Performance (satisfaction of an upper bound based on transport delay Eq. (6) on the test set of differently trained models—we report the average (over all nodes and scenarios) and standard deviation rounded to two decimal places

	Demands		Evaluation					
			Normal			Rand		
		Cl pattern(s)	Spike	Wave	Rand	Spike	Wave	Rand
Training	Normal	Spike	$1.0 {\scriptstyle \,\pm \,0.0 }$	$1.0 {\scriptstyle \,\pm \,0.0 }$	$1.0 {\scriptstyle \,\pm \,0.0 }$	$1.0 {\scriptstyle \,\pm \,0.0 }$	$1.0 {\scriptstyle \,\pm \,0.0 }$	$1.0 {\scriptstyle \,\pm \,0.0 }$
		Spike + Rand	$1.0 {\scriptstyle \,\pm \,0.0 }$	$0.97 {\scriptstyle \,\pm \,0.02 }$	$1.0 {\scriptstyle \,\pm \,0.0 }$	$1.0 {\scriptstyle \,\pm \,0.0 }$	$0.97 {\scriptstyle \,\pm \,0.03 }$	$1.0 {\scriptstyle \,\pm \,0.0 }$
	Rand	Spike	$1.0 {\scriptstyle \,\pm \,0.0 }$	$1.0 {\scriptstyle \,\pm \,0.0 }$	$1.0 {\scriptstyle \,\pm \,0.0 }$	$1.0 {\scriptstyle \,\pm \,0.0 }$	$1.0 {\scriptstyle \,\pm \,0.0 }$	$1.0 {\scriptstyle \,\pm \,0.0 }$
		Spike + Rand	$1.0 {\scriptstyle \,\pm \,0.0 }$	$0.98 {\scriptstyle \,\pm \,0.01 }$	$1.0 {\scriptstyle \,\pm \,0.0 }$	$1.0 {\scriptstyle \,\pm \,0.0 }$	$0.97 {\scriptstyle \,\pm \,0.02 }$	$1.0 {\scriptstyle \,\pm \,0.0 }$
	All	Spike + Rand	$1.0 {\scriptstyle \,\pm \,0.0 }$	$0.96 {\scriptstyle \,\pm \,0.02 }$	$1.0 {\scriptstyle \,\pm \,0.0 }$	$1.0 {\scriptstyle \,\pm \,0.0 }$	$0.97 {\scriptstyle \,\pm \,0.02 }$	$1.0 {\scriptstyle \,\pm \,0.0 }$

## Footnotes

1 https://github.com/andreartelt/A-Benchmark-for-Physics-informed-Machine-Learning-of-Chlorine-States-in-Water-Distribution-Networks.

2 https://waterfutures.github.io/WaterBenchmarkHub/benchmarks/ClStateEstimate.html.

3 https://github.com/andreartelt/A-Benchmark-for-Physics-informed-Machine-Learning-of-Chlorine-States-in-Water-Distribution-Networks.
